# Supplementary material for: KCTD15 acts as an anti-tumor factor in colorectal cancer cells downstream of the demethylase FTO and the m6A reader YTHDF2
Source: Commun Biol. 2024 Mar 4;7:262. doi: 10.1038/s42003-024-05880-9 (PMC10912199; doi:10.1038/s42003-024-05880-9)
Supplement: Supplementary file 2 — Supplementary information [file 42003_2024_5880_MOESM2_ESM.pdf]

Supplementary information

Supplementary Figures

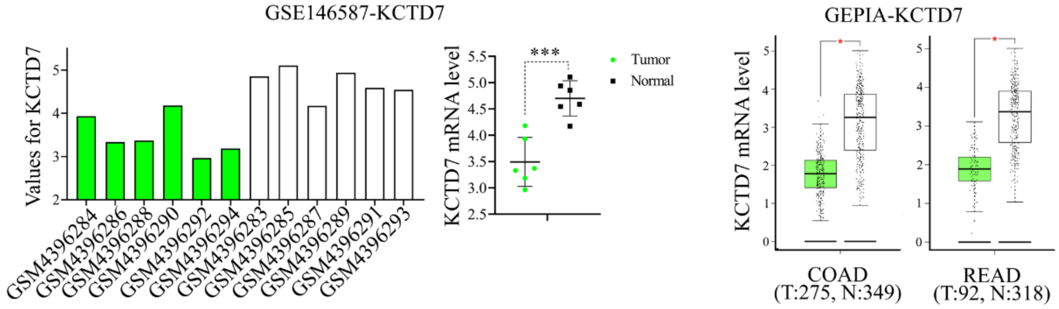

Supplementary Figure 1 The specific expression values of KCTD7 in the three CRC datasets.

*P < 0.05 and ***P < 0.001.

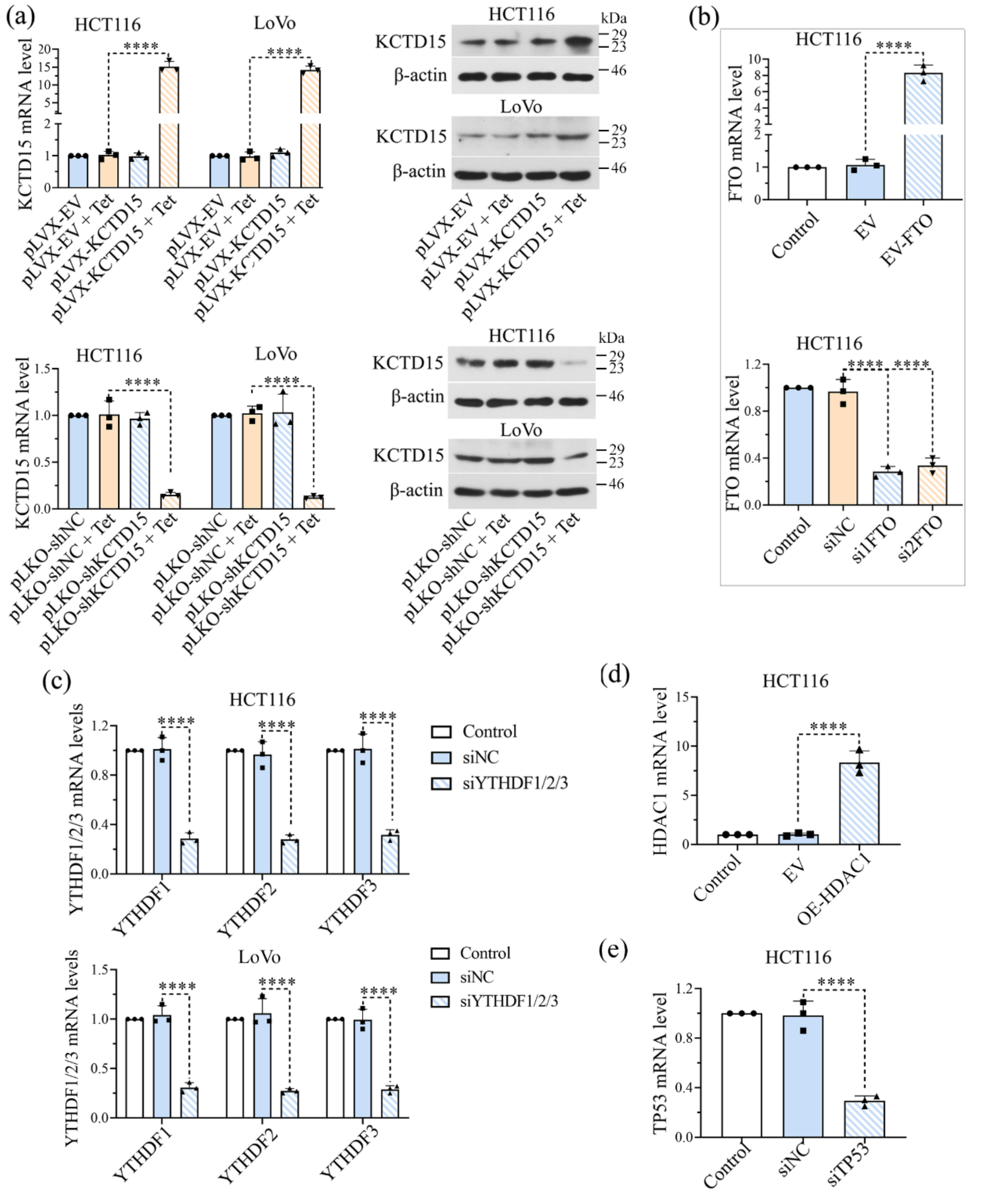

Supplementary Figure 2 Validation of transfection or infection efficiency. (a) KCTD15 mRNA expression was verified by qRT-PCR and Western Blot. **(b)** The mRNA expression levels of **(b)** FTO， **(c)** YTHDF1, 2, and 3, **(d)** HDAC1, and **(e)** TP53 were determined by qRT-PCR. ****P < 0.0001. n = 3 per group.

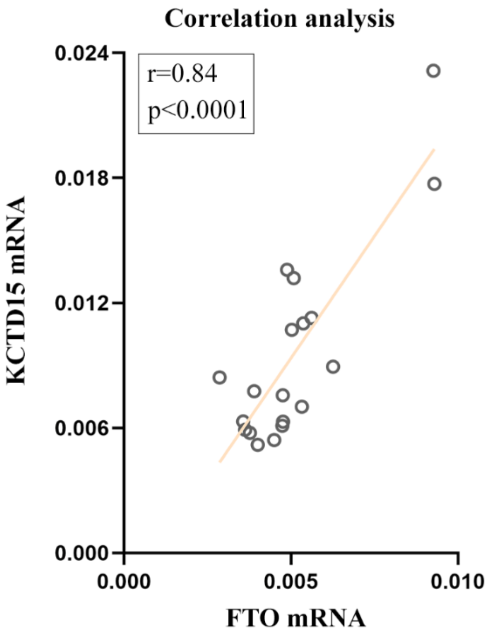

Supplementary Figure 3 The mRNA expression correlation between FTO and KCTD15 in CRC tissue samples.

Figure 3d

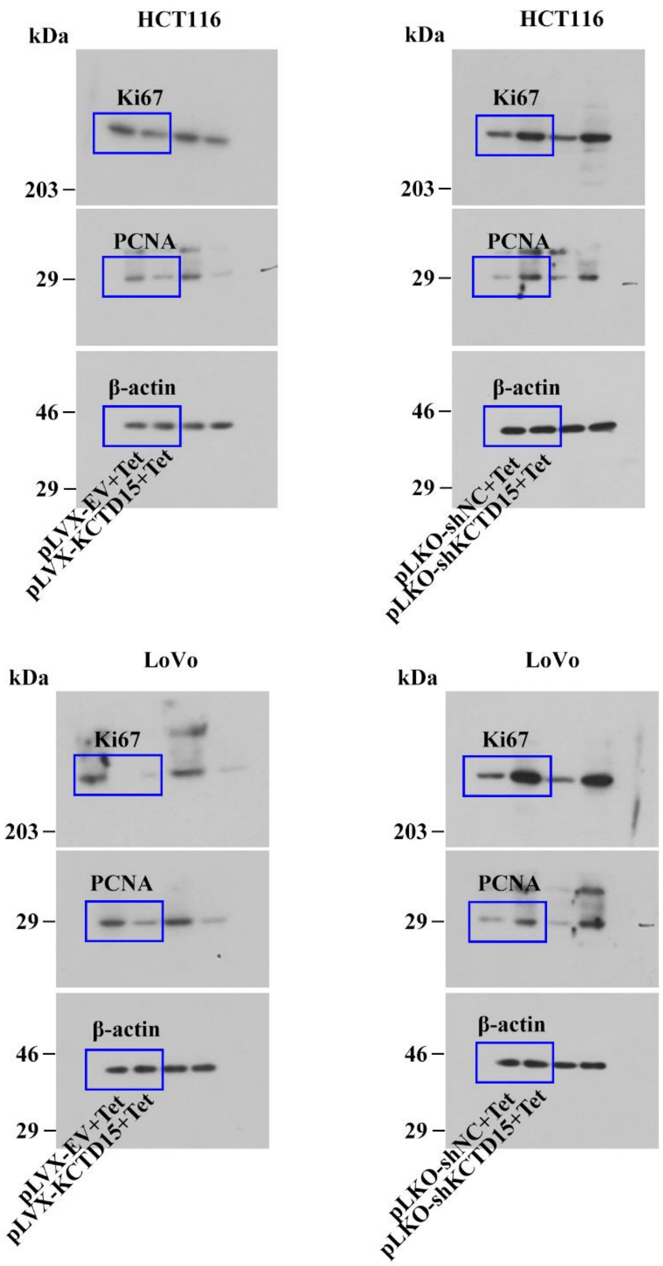

Supplementary Figure 4 Uncropped blot images in Figure 3.

Figure 4d

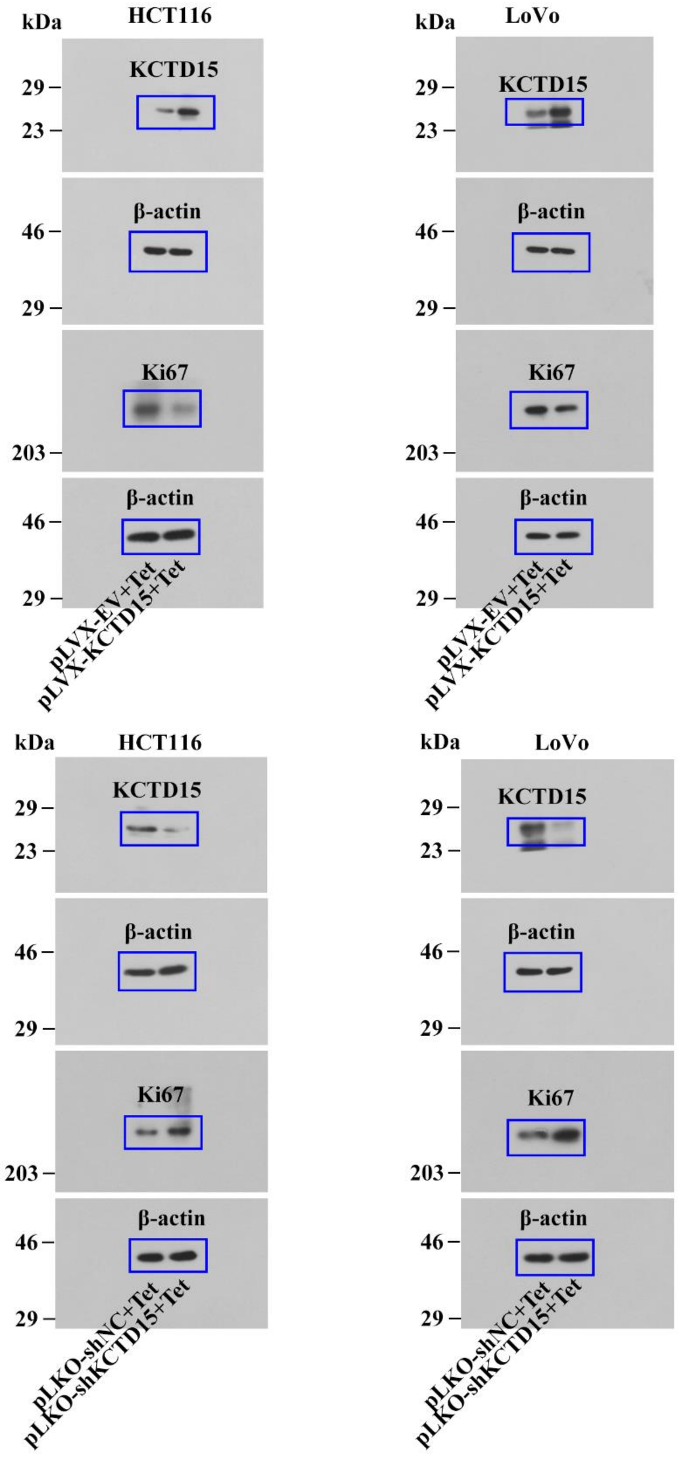

Supplementary Figure 5 Uncropped blot images in Figure 4.

Figure 5b

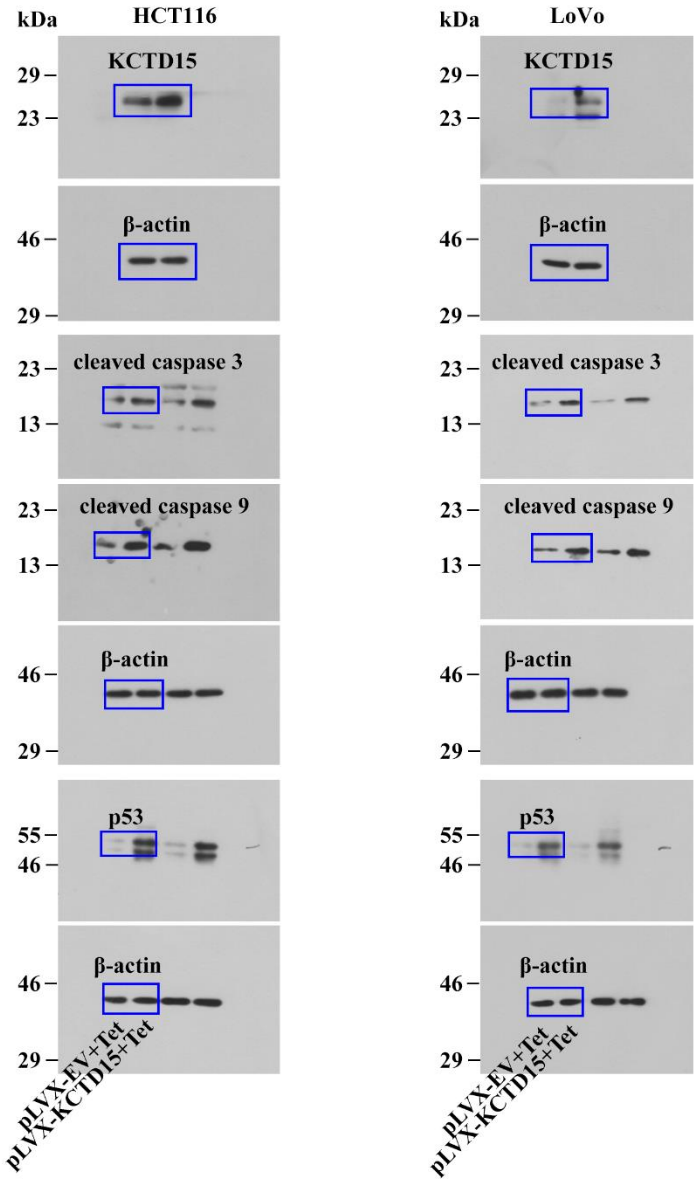

Supplementary Figure 6 Uncropped blot images in Figure 5.

Figure 7d

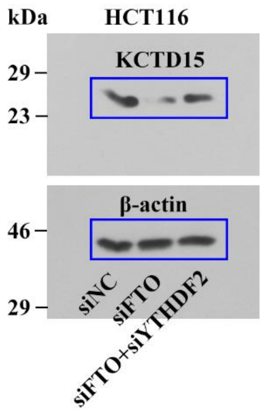

Figure 7e

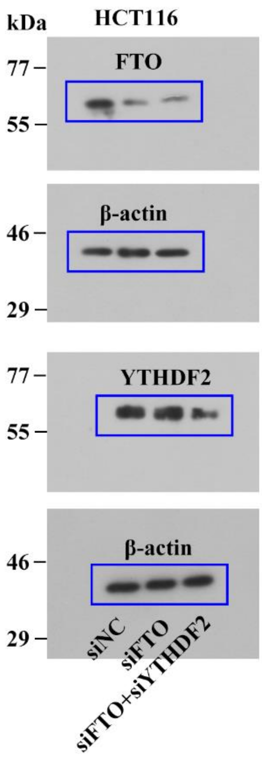

Supplementary Figure 7 Uncropped blot images in Figure 7.

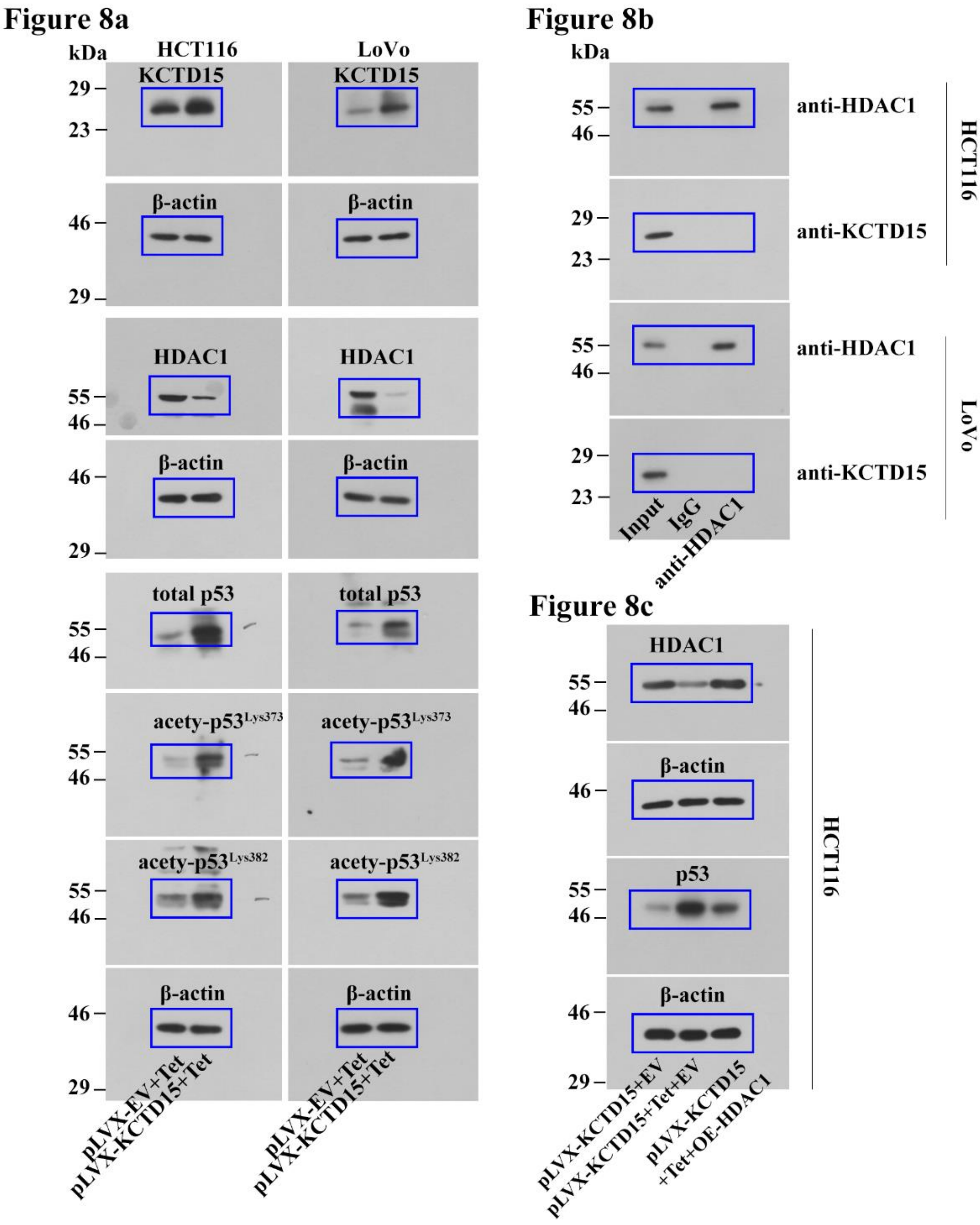

Supplementary Figure 8 Uncropped blot images in Figure 8.

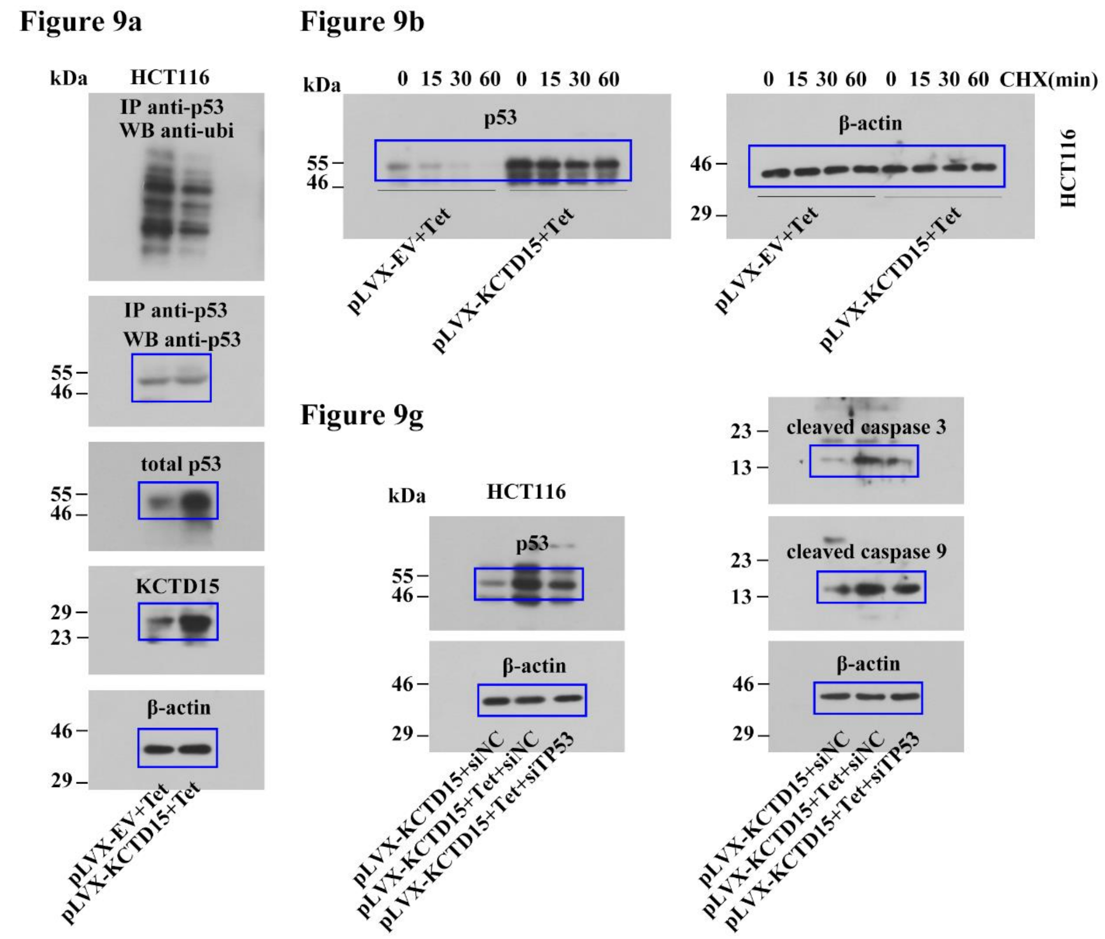

Supplementary Figure 9 Uncropped blot images in Figure 9.

Supplementary Figure 2a

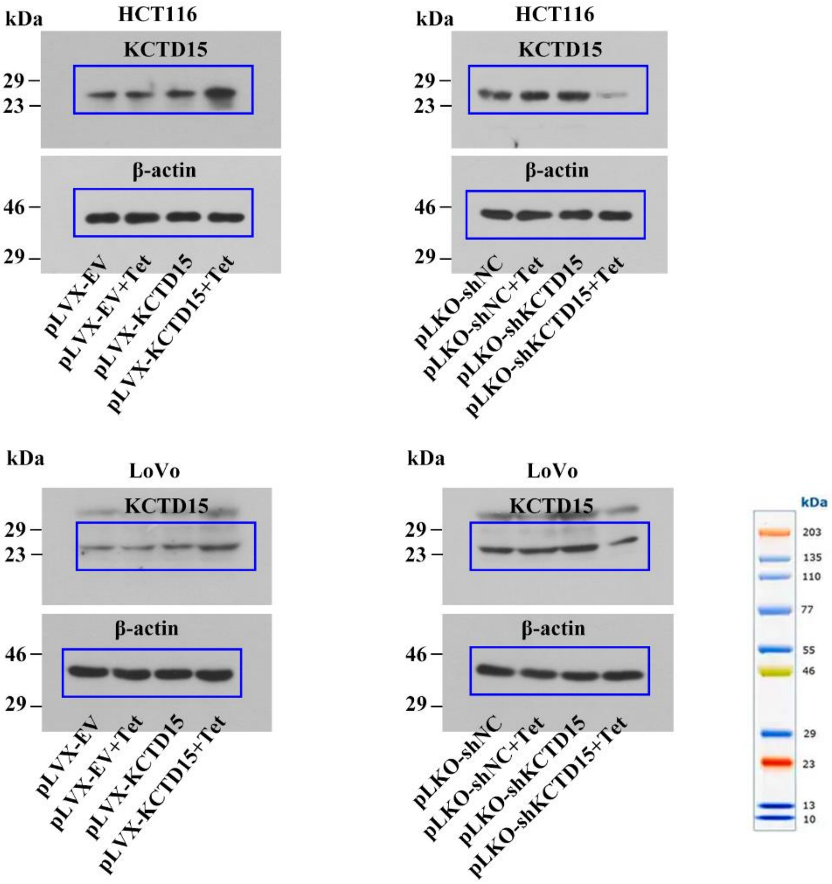

Supplementary Figure 10 Uncropped blot images in Supplementary Figure 2.

Supplementary Tables

Supplementary Table. S1 The sequences of shRNA and siRNA.

Name	Sequences (5'-3')
NC shRNA	Sense TTCTCCGAACGTGTCACGT (Targeting area)
KCTD15 shRNA	Sense GGATAAGCCGCCTCTTCAATG (Targeting area)
FTO siRNA-1	Sense GGUUAGGAUCCAAGGCAAATT
FTO siRNA-2	Sense GGAUGACUCUCAUCUCGAATT
TP53 siRNA	Sense GCUUCGAGAUGUUCCGAGATT
YTHDF1 siRNA	Sense ACAAGAUAAUAAAGUACAATT
YTHDF2 siRNA	Sense GCACAGAAGUUGCAAGCAATT
YTHDF3 siRNA	Sense GGUAUGACUAGCAUUGCAATT
NC siRNA	Sense UUCUCCGAACGUGUCACGUTT

Supplementary Table. S2 The primer sequences for qRT-PCR.

Gene name	Primer sequences (5'-3')
FTO	Forward GAACACCAGGCTCTTTACG
	Reverse ATGAACCCATCCCAACC
KCTD15	Forward GCGGAGGGAGGAAACAT
	Reverse TCAGGACGTAGCGGAAAA
YTHDF1	Forward CAATGAGGCTCCGTGGTC
	Reverse AAACAGCATCGTGCATAAAA
YTHDF2	Forward TTTGATGTCAGGTGGATTT
	Reverse TTGGCGTTTCTCATAGTG
YTHDF3	Forward TGCGTATGCTGGTGTCT
	Reverse GGCTTCCTCCTCTTCTT
β-actin	Forward GGCACCCAGCACAATGAA
	Reverse TAGAAGCATTTGCGGTGG
RIP-KCTD15	Forward TGCTTCAGTGCCCACCT
Primer 1	Reverse ATCCCACCGCTGTATCTTT
RIP-KCTD15	Forward TCTTGACCCCTGATTCC
Primer 2	Reverse CACAGGATGGTGATGGC

Supplementary Table. S3 The antibodies information for western blot.

Primary antibody	Dilution	Source	Catalog number
KCTD15	1:1000	Proteintech	20128-1-AP
Ki67	1:1000	Affinity	AF0198
PCNA	1:1000	Affinity	AF0239
cleaved caspase-3	1:1000	Affinity	AF7022
cleaved caspase-9	1:1000	Affinity	AF5240
p53	1:5000	Proteintech	60283-2-Ig
FTO	1:1000	ABclonal	A20992
HDAC1	1:1000	Affinity	AF6433
acety-p53 Lys373	1:5000	Abcam	ab62376
acety-p53 Lys382	1:1000	Abcam	ab75754
β-actin	1:1000	Santa cruz	sc-47778

Secondary antibody	Dilution	Source	Catalog number
Goat anti-rabbit IgG	1:1000	Beyotime	A0208
Goat anti-mouse IgG	1:1000	Beyotime	A0216

Supplementary Table. S4 The antibodies information for co-immunoprecipitation (co-IP) and ubiquitination assay.

Primary antibody	Source	Catalog number
P53	Proteintech	60283-2-Ig
HDAC1	Proteintech	10197-1-AP
Ubiqutin	Proteintech	10201-2-AP
Goat anti-rabbit IgG-HRP	Proteintech	SA00001-2
