# Supplementary material for: KCTD15 acts as an anti-tumor factor in colorectal cancer cells downstream of the demethylase FTO and the m6A reader YTHDF2
Source: Commun Biol. 2024 Mar 4;7:262. doi: 10.1038/s42003-024-05880-9 (PMC10912199; doi:10.1038/s42003-024-05880-9)
Supplement: Supplementary file 3 — Description of Additional Supplementary Files [file 42003_2024_5880_MOESM3_ESM.pdf]

Description of Additional Supplementary Files

File Name: Supplementary Data 1

Description: The source data for the Figures.
