# Supplementary material for: KCTD15 acts as an anti-tumor factor in colorectal cancer cells downstream of the demethylase FTO and the m6A reader YTHDF2
Source: Commun Biol. 2024 Mar 4;7:262. doi: 10.1038/s42003-024-05880-9 (PMC10912199; doi:10.1038/s42003-024-05880-9)
Supplement: Supplementary file 5 — Reporting summary [file 42003_2024_5880_MOESM5_ESM.pdf]

nature portfolio

Corresponding author(s): Tie-Ning Zhang, Huan-Huan Chen

Last updated by author(s): Dec 26, 2023

Reporting Summary

Nature Portfolio wishes to improve the reproducibility of the work that we publish. This form provides structure for consistency and transparency in reporting. For further information on Nature Portfolio policies, see our Editorial Policies and the Editorial Policy Checklist.

Statistics

For all statistical analyses, confirm that the following items are present in the figure legend, table legend, main text, or Methods section.

n/a	Confirmed	
☐	☒	The exact sample size (n) for each experimental group/condition, given as a discrete number and unit of measurement
☐	☒	A statement on whether measurements were taken from distinct samples or whether the same sample was measured repeatedly
☐	☒	The statistical test(s) used AND whether they are one- or two-sided<br>*Only common tests should be described solely by name; describe more complex techniques in the Methods section.*
☒	☐	A description of all covariates tested
☐	☒	A description of any assumptions or corrections, such as tests of normality and adjustment for multiple comparisons
☐	☒	A full description of the statistical parameters including central tendency (e.g. means) or other basic estimates (e.g. regression coefficient) AND variation (e.g. standard deviation) or associated estimates of uncertainty (e.g. confidence intervals)
☐	☒	For null hypothesis testing, the test statistic (e.g. F, t, r) with confidence intervals, effect sizes, degrees of freedom and P value noted<br>*Give P values as exact values whenever suitable.*
☒	☐	For Bayesian analysis, information on the choice of priors and Markov chain Monte Carlo settings
☒	☐	For hierarchical and complex designs, identification of the appropriate level for tests and full reporting of outcomes
☒	☐	Estimates of effect sizes (e.g. Cohen's d, Pearson's r), indicating how they were calculated

Our web collection on statistics for biologists contains articles on many of the points above.

Software and code

Policy information about availability of computer code

Data collection	A CRC gene expression profile (GSE146587) was downloaded from Gene Expression Omnibus (GEO). Two datasets regarding colon adenocarcinoma (COAD) and rectum adenocarcinoma (READ) from The Cancer Genome Atlas (TCGA) were analyzed via GEPIA.
Data analysis	GraphPad Prism 8.0.1 (GraphPad Software Inc., San Diego, CA, USA) software.

For manuscripts utilizing custom algorithms or software that are central to the research but not yet described in published literature, software must be made available to editors and reviewers. We strongly encourage code deposition in a community repository (e.g. GitHub). See the Nature Portfolio guidelines for submitting code & software for further information.

Data

Policy information about availability of data

All manuscripts must include a data availability statement. This statement should provide the following information, where applicable:

- Accession codes, unique identifiers, or web links for publicly available datasets
- A description of any restrictions on data availability
- For clinical datasets or third party data, please ensure that the statement adheres to our policy

The source data underlying the graphs in this study are available at Supplementary Data 1. The uncropped gel images are provided in Supplementary Figures 4-10. All other data are available from the corresponding author on reasonable request.

Research involving human participants, their data, or biological material

Policy information about studies with human participants or human data. See also policy information about sex, gender (identity/presentation), and sexual orientation and race, ethnicity and racism.

Reporting on sex and gender	Specific information on the clinical sample has been provided in Table 1, including statistics on patient gender.
Reporting on race, ethnicity, or other socially relevant groupings	We don't have related issues.
Population characteristics	Specific information on the clinical sample has been provided in Table 1.
Recruitment	CRC specimens (86 males and 39 females, median age 59.5, range 26-81) and nineteen fresh paired normal and CRC specimens (9 males and 10 females, median age 67, range 56-86) were collected.
Ethics oversight	All experiments were approved by the Ethics Committee of Shengjing Hospital of China Medical University.

Note that full information on the approval of the study protocol must also be provided in the manuscript.

Field-specific reporting

Please select the one below that is the best fit for your research. If you are not sure, read the appropriate sections before making your selection.

☒ Life sciences ☐ Behavioural & social sciences ☐ Ecological, evolutionary & environmental sciences

For a reference copy of the document with all sections, see nature.com/documents/nr-reporting-summary-flat.pdf

Life sciences study design

All studies must disclose on these points even when the disclosure is negative.

Sample size	mice experiments: sample size = 6; cell experiments: sample size = 3.
Data exclusions	No data were excluded.
Replication	All experiments were performed at least three times.
Randomization	The sample was randomly assigned.
Blinding	Each experiment was associated with proper controls, and compared samples were collected and analyzed under the same conditions.

Reporting for specific materials, systems and methods

We require information from authors about some types of materials, experimental systems and methods used in many studies. Here, indicate whether each material, system or method listed is relevant to your study. If you are not sure if a list item applies to your research, read the appropriate section before selecting a response.

Materials & experimental systems

n/a	Involved in the study	
☐	☒	Antibodies
☐	☒	Eukaryotic cell lines
☒	☐	Palaeontology and archaeology
☐	☒	Animals and other organisms
☐	☒	Clinical data
☒	☐	Dual use research of concern
☒	☐	Plants

Methods

n/a	Involved in the study	
☒	☐	ChIP-seq
☐	☒	Flow cytometry
☒	☐	MRI-based neuroimaging

Antibodies

Antibodies used	KCTD15, Proteintech (Catalog number: 20128-1-AP)<br>Ki67, Affinity (Catalog number: AF0198)<br>PCNA, Affinity (Catalog number: AF0239)

	HDAC1, Affinity (Catalog number:AF6433)<br>cleaved caspase-3, Affinity (Catalog number: AF7022)<br>cleaved caspase-9, Affinity (Catalog number: AF5240)<br>p53, Proteintech (Catalog number: 60283-2-Ig)<br>FTO, ABclonal (Catalog number: A20992)<br>acety-p53 Lys373, Abcam (Catalog number: ab62376)<br>acety-p53 Lys382, Abcam (Catalog number: ab75754)<br>β-actin, Santa cruz (Catalog number: sc-47778)
Validation	No validation for commercial antibodies.

Eukaryotic cell lines

Policy information about cell lines and Sex and Gender in Research

Cell line source(s)	The CRC cell lines (HCT116 and LoVo) were purchased from iCell (Shanghai, China).
Authentication	STR authentication from the manufacturers.
Mycoplasma contamination	We state that no mycoplasma contamination tests were performed on the cell line.
Commonly misidentified lines (See ICLAC register)	No commonly misidentified lines.

Animals and other research organisms

Policy information about studies involving animals; ARRIVE guidelines recommended for reporting animal research, and Sex and Gender in Research

Laboratory animals	Male BALB/c nude mice were used for this study.
Wild animals	No wild animals were used in this study.
Reporting on sex	No effects of different sex animals on experimental studies.
Field-collected samples	No field-collected samples were used in this study.
Ethics oversight	All animal experimental procedures were approved by Ethical Committee of Shengjing Hospital of China Medical University.

Note that full information on the approval of the study protocol must also be provided in the manuscript.

Clinical data

Policy information about clinical studies

All manuscripts should comply with the ICMJE guidelines for publication of clinical research and a completed CONSORT checklist must be included with all submissions.

Clinical trial registration	N/A
Study protocol	Cancer and paracancer samples were collected to detect KCDT15 expression.
Data collection	The samples were collected from colorectal cancer patients admitted to Shengjing Hospital of China Medical University.
Outcomes	The expression of KCTD15 was negatively correlated with clinicopathological factors of colorectal cancer.

Flow Cytometry

Plots

Confirm that:

☒ The axis labels state the marker and fluorochrome used (e.g. CD4-FITC).

☒ The axis scales are clearly visible. Include numbers along axes only for bottom left plot of group (a 'group' is an analysis of identical markers).

☒ All plots are contour plots with outliers or pseudocolor plots.

☒ A numerical value for number of cells or percentage (with statistics) is provided.

Methodology

Sample preparation	Cells were seeded into 6-well plates at density of 5×105 cells each well. After infection or transfection, cells were harvested

Sample preparation	and washed twice with PBS and resuspended in binding buffer (500 µL). Then, cells were stained with Annexin V–FITC (5 µL, KeyGEN BioTECH) and PI (5 µL, KeyGEN BioTECH) for 10 min in the dark.
Instrument	flow cytometer (Aceabio, San Diego, CA, USA)
Software	FlowJo v10.8.0
Cell population abundance	The proportion of the total number of early apoptotic cells and late apoptotic cells indicates the rate of apoptosis.
Gating strategy	We selected more than 90% of the cell populations as the gating strategy and as the preliminary FSC/SSC gates of the starting cell population.

☒ Tick this box to confirm that a figure exemplifying the gating strategy is provided in the Supplementary Information.
